# Economic and other barriers to adopting recommendations to prevent childhood obesity: results of a focus group study with parents

**DOI:** 10.1186/1471-2431-9-81

**Published:** 2009-12-21

**Authors:** Kendrin R Sonneville, Nancy La Pelle, Elsie M Taveras, Matthew W Gillman, Lisa A Prosser

**Affiliations:** 1 Department of Nutrition, Harvard School of Public Health, Boston, MA, USA; 2 Children's Hospital, Boston, Boston, MA, USA; 3 Preventive and Behavioral Medicine, University of Massachusetts Medical School, Worcester, MA, USA; 4 Obesity Prevention Program, Department of Population Medicine, Harvard Medical School and Harvard Pilgrim Health Care, Boston, MA, USA; 5 Division of General Pediatrics, Children's Hospital Boston, Boston, MA, USA; 6 Child Health Evaluation and Research Unit, Division of General Pediatrics, University of Michigan Health System, Ann Arbor, MI, USA

## Abstract

**Background:**

Parents are integral to the implementation of obesity prevention and management recommendations for children. Exploration of barriers to and facilitators of parental decisions to adopt obesity prevention recommendations will inform future efforts to reduce childhood obesity.

**Methods:**

We conducted 4 focus groups (2 English, 2 Spanish) among a total of 19 parents of overweight (BMI ≥ 85th percentile) children aged 5-17 years. The main discussion focused on 7 common obesity prevention recommendations: reducing television (TV) watching, removing TV from child's bedroom, increasing physically active games, participating in community or school-based athletics, walking to school, walking more in general, and eating less fast food. Parents were asked to discuss what factors would make each recommendation more difficult (barriers) or easier (facilitators) to follow. Participants were also asked about the relative importance of economic (time and dollar costs/savings) barriers and facilitators if these were not brought into the discussion unprompted.

**Results:**

Parents identified many barriers but few facilitators to adopting obesity prevention recommendations for their children. Members of all groups identified economic barriers (time and dollar costs) among a variety of pertinent barriers, although the discussion of dollar costs often required prompting. Parents cited other barriers including child preference, difficulty with changing habits, lack of information, lack of transportation, difficulty with monitoring child behavior, need for assistance from family members, parity with other family members, and neighborhood walking safety. Facilitators identified included access to physical activity programs, availability of alternatives to fast food and TV which are acceptable to the child, enlisting outside support, dietary information, involving the child, setting limits, making behavior changes gradually, and parental change in shopping behaviors and own eating behaviors.

**Conclusions:**

Parents identify numerous barriers to adopting obesity prevention recommendations, most notably child and family preferences and resistance to change, but also economic barriers. Intervention programs should consider the context of family priorities and how to overcome barriers and make use of relevant facilitators during program development.

## Background

Childhood obesity is a prominent public health concern that is associated with an increased risk of cardiovascular disease, diabetes, some cancers, depression, discrimination and weight-related bias, and other morbidities [1]. The short- and long-term risks to both physical and psychological health of childhood obesity translate into a reduced quality of life [2-5]. As such, intervening early may be necessary to reduce immediate consequences of childhood obesity and engender healthy behaviors before obesogenic lifestyle patterns are deeply established [6,7].

Parents control many aspects of the nutrition and physical activity environment that contribute to a child's health-related behaviors [8-10]. However, our understanding of the range of barriers parents face when adopting recommendations to prevent childhood obesity, and the relative importance of these barriers, is incomplete. Focus group studies with parents have identified barriers to adopting recommendations to increase physical activity which include weather, siblings, finances, time, neighborhood safety, child's preferences for sedentary activities, lack of affordable and accessible recreation facilities, and low caregiver motivation [11-14]. Findings from two focus group studies suggest that parents believe the external environmental and social barriers hold responsibility for some of children's unhealthful eating habits, beliefs which may impact parental receptiveness to interventions aimed at the family level [15,16].

Economic considerations, such as the high cost of nutritious foods and affordability of junk foods and fast foods, are commonly assumed determinants of food choice, in part because low-income families exhibit among the highest prevalence of childhood obesity in the U.S. [17]. In considering economic barriers, however, it is important to consider both out-of-pocket dollar costs and time costs. For example, reducing fast food meals could reduce food costs but would require additional time for food preparation (a decrease in dollar costs but an increase in time costs). If a child enrolled in a sports program, a parent may be required to pay enrollment fees or purchase equipment, as well as invest in additional transportation time (an increase in both dollar costs and time costs).

An emerging literature is focusing on the economics of obesity in adults, but there has been less focus on the economics of childhood obesity. MacInnis & Rausser suggest a role for energy-dense processed foods, which have become less expensive, in contributing to childhood obesity or risk of obesity [18]. A related theme of the impact of technological change in food production on adult obesity has been explored by Cutler et al. [19]. Anderson et al. examined whether the likelihood of a child being overweight is linked to mothers entering the workforce, but only found an effect for upper income families [20]. A possible explanation for this perplexing result is that lower income mothers may be too time-constrained to focus on their child's nutrition and physical activity whether or not they work outside the home. This supports the hypothesis that economic constraints (time and dollar) may be especially pertinent to families with children at higher risk for obesity, those of lower socio-economic status.

Obesity prevention and management recommendations for children involve changes in parental behavior. As such, understanding parental decision-making related to adopting recommendations is critical and could inform future efforts to reduce childhood obesity. For example, information about parental barriers and facilitators could lead to obesity interventions that are tailored to maximize parental compliance.

The objective of this study was to explore, through the use of qualitative methods, barriers and facilitators that influence parenting behaviors and decisions that relate to child food choices, activities, and other behaviors that could affect a child's risk of obesity. We focused especially on economic barriers, including both time and dollar costs, an understudied yet potentially salient barrier. Focus groups are conducive to identifying constructs of potential interest for future research and in determining whether non-economic barriers generate as much discussion as economic ones.

## Methods

We held four focus groups in Boston, MA, during October and November 2007: two in English and two in Spanish. We recruited parents of overweight children (BMI-for-age ≥ 85th percentile) aged 5-17 years attending the Preventive Cardiology clinic at Children's Hospital Boston or one of two weight management clinics at the hospital, One Step Ahead and Optimal Weight for Life (n = 19). Three pairs of the English-speaking participants in one focus group were married partners; therefore, data relate to 16 children. Focus group size ranges from 2 to 7 parents. Additional demographic data about the parent or the family were not collected at the focus groups.

Focus groups lasted two hours. Parents received a $40 gift card incentive for participating in the focus groups. Dinner was provided at each focus group, as was a parking or taxi voucher. Participants completed written informed consent prior to the start of the focus group. The study protocol was approved by the Institutional Review Boards of Children's Hospital Boston and Harvard Pilgrim Health Care.

A professional focus group facilitator (non-physician), who was bilingual in Spanish and English, conducted all four focus groups. The group facilitator used a semi-structured interview guide for the focus groups that began with an ice-breaker activity and a brief warm-up exercise. The main discussion focused on common obesity prevention recommendations selected by the authors: reducing TV watching time, removing TV from child's bedroom, increasing time spent playing physically active games, participating in community or school-based athletics, walking to and from school, walking more in general, and eating fewer fast food meals. Parents were asked to assess how difficult or easy each type of advice was for them to follow and to discuss what factors would make each type of advice more difficult (barriers) or easier (facilitators) to implement. Participants were also asked about the relative importance of economic (time and dollar costs/savings) barriers and facilitators if these were not brought into the discussion unprompted.

We audiotaped and transcribed all focus groups to facilitate analysis and translated Spanish groups into English during transcription. Data were then transcribed into a Microsoft Word table with columns for focus group identifier, theme code, speaker ID, speaker comment and chronological sequence number [21]. We conducted qualitative analysis using the template organizing style [22-24]. The qualitative research analyst (NL) read all transcripts in detail and constructed a theme codebook that documented themes and subthemes in a logical hierarchy and assigned a numerical theme code to each theme and subtheme to facilitate sorting.

We completed a topic by topic thematic analysis across focus groups within each population segment, defined as English-speaking and Spanish-speaking. Comparison tables with six columns, one for theme code, one for theme description, and one each for the four focus groups, were created and notations were made whenever one or more speakers in each focus group voiced a specific theme. After completing these notations for both English and Spanish speaking focus groups, themes were re-ordered for each focus group major theme in the comparison table such that subthemes that were voiced more frequently occur in an earlier row of the table. For each theme grouping across the four focus groups, we completed a comparative analysis and comparative cross-population segment thematic analysis.

Validation was done after preliminary coding. All similarly coded text was reviewed to ensure that text that was not representative of the category definition was not included. Where errors were detected, the inappropriate text was recoded as an instance of the more appropriate theme and regrouped with text representing the corrected theme for data reduction.

During data reduction, a table was created with one column per each of the four focus groups and one row for each theme code. In each cell, the number of participants within each focus group who explicitly mentioned that particular theme or subtheme in a similar way was recorded along with the participants' initials so that multiple similar responses from the same participant were not overcounted. Themes could then be prioritized by frequency of mentions both within and across focus groups.

## Results

When asked about the importance of healthy weight compared to other childhood health issues during the warm-up exercise, some parents felt that child safety and academic performance were of higher priority than obesity prevention. Parents went on to identify various barriers and facilitators to adopting obesity prevention recommendations throughout the focus group discussion (Table 1). Although "providing a healthier diet" was not one of the recommendations posed by the moderator explicitly, it was raised by participants themselves as advice they often hear. Barriers and facilitators to providing a healthier diet voiced by parents are also included in Table 1.

**Table 1:** Barriers and facilitators to adopting obesity prevention recommendations cited by parents

Obesity Prevention Recommendation	Barrier	Facilitator
Reduce time watching TV	Monitoring/Time cost	Availability of acceptable alternatives

Remove TV from child's bedroom	Difficulty with changing habits Parity with other family members	Early limit setting

Increase physical activity	Time cost Lack of information Lack of transportation Child preference Safety Dollar cost Need assistance from family members	Availability of acceptable alternatives Enlist outside support

Participate in school/community-based sports	Practicality/Time cost Child preference	Access

Walk to school	Safety	None identified

Walk more in general	Child preference	None identified

Eat less fast food	Need assistance from family members Child preference/Convenience Time cost	Information about dollar costs Parental behavior change

Provide a healthier diet	Parity with other family members Dollar cost Child preference Difficulty with changing habits	Parental behavior change Involve the child Limit setting Make behavior changes gradually

### Barriers

The barriers most frequently voiced regarding reducing time watching TV related to monitoring/time costs. After being asked what their children do when they are not watching TV at home, 2 parents in one focus group responded "driving me crazy" and one parent responded "fighting with his little sister". The barriers most frequently voiced regarding removing TVs from a child's bedroom related to difficulty with changing habits ("They've had TV since they've had their own room") and parity with other family members ("For me it is kind of hard for me to take the TV out of her bedroom when I have one in my bedroom, you know.")

The barriers most frequently voiced regarding increasing physical activity related to time costs ("I have a hard time with extracurricular sports activities and homework, because I felt like I couldn't do it all"; "I would have to make compromises in my life, to be able to, say, put him into lacrosse, which is a fabulous activity and I'd love to do it, but I just can't figure out how to do that... timewise. It takes a lot of my time."), lack of information (parents lack information about locations where they could engage in physical activity), lack of transportation ("If there was transportation where I live, I would get on the bus and go to a playground with my girls."), child preference ("She doesn't like to play like basketball, nothing like that."), safety ("If he's home playing video games as opposed to being out, I almost feel like he's safer even though, probably, mentally it's not"), dollar costs ("Hockey is very expensive, football is very expensive, you know, in terms of the equipment. Maybe not so much in terms of the fee, but all of the equipment involved."), and the need for assistance from family members (parents lack help from extended family to get children to locations where they could engage in physical activity). The barrier most frequently voiced related to participating in school/community-based sports was child preference ("Football, hockey and baseball are... very popular. He doesn't like them. He doesn't have an interest in joining a group team.").

The barriers most frequently voiced regarding walking to school related to practicality/time cost ("School is about 15 miles away, and the bus stop is only around the corner from our house"; "His school is actually like 2 minutes away, for a start. I'm on my way to work and I just drop him off, so it's not a necessity for him to walk.") and safety ("Going down a busy road is too dangerous for them. Just the security, of someone driving by and being able to grab them, I wouldn't let them do it. And if I have them taking the bus, I know where they are before and after school."). The barrier most frequently voiced regarding walking more in general was child preference ("She doesn't like to walk... She likes her computer.").

The barriers most frequently voiced regarding eating less fast food related to the need for assistance from other family members ("Nana is going to give them whatever they want. Soda. Because I don't give my kids soda... And, I mean, she sneaks it to them."), child preference ("Going to McDonalds is like a treat for them. She loves to go to McDonalds... It's like the most important thing to go to McDonald*.*"), and time costs ("It is difficult for me to eat fewer fast food meals because I work two shifts, so it's like easier for me to buy them something to eat.").

The barriers most frequently voiced regarding providing a healthier diet included parity with other family members ("If I feel like having junk food, she's going to have it too. I can't say that I'm going to have it for myself and not let her have it"; " For me it is a little difficult because my 19-year-old daughter is very skinny. So my problem is they each have their own list. This one wants one thing and the other wants something else."), dollar costs ("When you have a large family to feed, you know, doctor's advice is "eat healthy", but everything that's healthy is very expensive."), and difficulty with changing habits (hard to change child's whole diet at once).

### Facilitators

In general, far fewer facilitators than barriers were identified for the obesity prevention recommendations. The facilitator most frequently voiced for reducing time watching TV was the availability of alternative activities. For the recommendation to remove TV from child's bedroom, the most frequently voiced facilitator related to early limit setting (tell child they cannot have a TV before they become teenagers). The facilitators most frequently voiced regarding increasing physical activity included availability of acceptable alternatives (new active video games that promote exercise inside; make physical activity inside the house more fun) and enlisting outside support (connecting children with Big Brothers or Big Sisters who might role model being physically active). The most frequent facilitator about increasing participation in school/community-based sports was access (need more easy-access community centers/local programs that provide physical activity opportunities). For reducing fast food, facilitators most frequently voiced included information about dollar costs (realizing that it costs less to cook a meal than to go out to eat) and parental behavior change (parents have to stop taking kids to fast food restaurants and buying fast food as takeout). The facilitators related to providing a healthier diet included parental behavior change ("I try not to buy like chips and stuff like that, because I know if it's there she's going to eat it."; "I've had bad eating habits for some time. I have to change my lifestyle and my way of eating in order to - and, like I said, I have made some changes, but there are things that I still need to work on."), involve the child (explain to child why diet needs to be changed and present with healthy options), limit setting ("I don't give them options."), and making behavior changes gradually ("Well they wanted me to stop everything at once, but I just took it one at a time. The first step was we stopped all the fried foods. Then we started eating more vegetables.").

### Cultural Issues Related to Weight and Food

Parents in all groups voiced cultural or inherited family beliefs/behaviors about food. Parents in the Spanish-speaking groups indicated that country of origin may influence food choices. Cultural issues regarding the perceptions of weight were voiced only in the Spanish-speaking groups. These included beliefs that being fat or large is "pretty" and that thinness is related to sickness. Parents in the Spanish-speaking groups said that grandparents don't believe that excess weight is unhealthy ("In my house the fatter one was the prettier one.").

### Economic vs. non-Economic Barriers

Economic considerations relating to both dollar and time costs were voiced as barriers to implementing several types of advice. However, parents cited non-economic barriers and time cost barriers more frequently in the focus group discussion than dollar cost barriers and dollar cost barriers were sometimes identified only after prompting. Economic barriers were sometimes cited by parents in conjunction with other barriers ("They recommended a few [cereals] she should be eating. It's more expensive. My daughter hates it. She says it's horrible.").

We asked parents in two focus groups how their lives would have to change in order to implement nutrition and physical activity recommendations. Changes that would necessitate time costs were identified. These included cutting back on work/study time to allow parents who worked or attended school to be home more to monitor child eating behaviors and spend more time with them ("For me, I also was thinking about making change in my job, so I can be home - to know that I get off at 5 o'clock... So then I can be home to monitor exactly what they eat. I don't know - if I'm at work until 8 o'clock, I don't know if they - they could be up eating M & Ms.").

## Discussion

Parents in this study identified numerous barriers and some facilitators to adopting obesity prevention recommendations. Barriers such as child resistance due to preference for existing behaviors and family member-related barriers were common. Parents acknowledged that expecting all family members to adopt recommendations was a considerable obstacle. Parents cited difficulties related to changing their own behaviors (such removing TV from their bedroom) and expressed ambivalence about enforcing recommendations that they themselves do not follow. Parents within the focus groups acknowledged that successful obesity prevention requires making family-based changes. Parents indicated that other family members may not support their efforts to adopt changes related to their child's weight and identified conflicting dietary needs of other children as a barrier, again highlighting the importance of the acceptability of behavior change within the context of the family and household decision-making model. Parents identified few facilitators to implementing recommendations. Parents recognized that changing their own eating habits and shopping habits would help facilitate their child's adherence to recommendations and acknowledged the role of parenting skills such as limit setting. Parents indicated that access to physical activity programs and the availability of alternatives to fast food and TV which are acceptable to the child would facilitate adherence.

Parents identified time cost barriers such as time required for transportation, to prepare healthy foods, to allow for participation in physical activity programs, and for behavior monitoring, more frequently than dollar cost barriers. Although parents identified dollar costs as barriers in all groups, they were not cited as the most important barriers. This may reflect the relative importance of dollar cost barriers or may have resulted because discussions of finances may be considered too sensitive or personal to be brought up without prompting. Further, dollar costs are not integral to some behavior changes such as removing the TV from the child's bedroom and walking to school so should not be a universally cited barrier. Additional research in this area should include individual survey research in which respondents may feel more comfortable discussing sensitive topics such as financial considerations. The frequency with which dollar costs are cited may also suggest that the influence of other barriers, such as child preference or family member-related barriers, dominate decisions about implementing recommendations. For example, parents may be less willing to make changes that involve time or dollar costs if their children are highly resistant to the changes.

This study was subject to some limitations. Due to recruiting challenges, the size of the focus groups varied (from 2 to 7 participants), the sample of Spanish-speaking parents was smaller than the sample of English-speaking parents, and the groups consisted of parents of children of all ages (the original study design called for stratifying focus groups by age). The small sample size limits the analysis of our findings as they may not be representative of the barriers/facilitators faced by all parents. Further, detailed demographic data about the parent and the family such as parental BMI and family size were not collected. Thus, differences in parental response based on parental or family characteristics were not assessed. Additional research assessing barriers among a large and varied sample of parents is warranted to explore the relationship between family characteristics and barriers faced. In addition, this study explored barriers and facilitators to implementing 7 specific types of obesity prevention recommendations. Although these represent common recommendations made by pediatricians, other possible recommendations related to nutrition and physical activity for overweight children were not included.

Given the limited number of studies that have considered economic factors contributing to physical activity and nutrition choices for children, this study adds to the literature by identifying both time and dollar costs as potential barriers to the adoption of obesity prevention. Further, this study identified that barriers related to all family members including children, parents, and other family members may prevail over these economic barriers. Future studies should consider the relative importance of the types of identified barriers and how barriers interact. In addition, ongoing research which examines how the entire family participates in decisions about nutrition and physical is needed. Interventions aimed at preventing obesity in childhood should be designed to help parents overcome barriers identified. For example, parents should be given support for changing their own behaviors and parents should be equipped to address issues such as child resistance and lack of support from other family members.

## Conclusions

The barriers identified in this study were numerous and varied, suggesting that parents face a unique combination of barriers when adopting recommendations to reduce childhood obesity. Economic considerations, especially time costs, may play a role in parental decisions to implement obesity-related recommendations and should be considered in the context of other priorities for the child and the family. Barriers should be routinely assessed and addressed when obesity-related recommendations are offered to enhance parental compliance. Future research should explore the extent to which identified barriers, including the needs and preferences of other family members, child preference, and economic considerations, influence parental decision making and should aim to identify approaches to surmounting these barriers.

## Competing interests

The authors declare that they have no competing interests.

## Authors' contributions

KS participated in the design and coordination of the study and helped to draft the manuscript. NL participated in the design of the study and conducted all qualitative analyses. ET and MG helped conceive of the study and participated in its design. LP conceived of the study, participated in its design, and helped to draft the manuscript. All authors read and approved the final manuscript.

## Pre-publication history

The pre-publication history for this paper can be accessed here:

http://www.biomedcentral.com/1471-2431/9/81/prepub
